# Evolution Under Pressure: Navigating Adaptation from Deep Time to the Anthropocene

**DOI:** 10.3390/biology15110888

**Published:** 2026-06-04

**Authors:** Mario Pagano, Sonia Del Prete

**Affiliations:** 1 Institute of Research on Terrestrial Ecosystems (IRET), National Research Council (CNR), Via Madonna del Piano 10, 50019 Sesto Fiorentino, Italy; 2 Institute of Biosciences and Bioresources (IBBR), National Research Council (CNR), Via Pietro Castellino 111, 80131 Naples, Italy; sonia.delprete@cnr.it

## 1. Introduction

Environmental stress has always represented one of the most powerful evolutionary forces determining biological diversity. Organisms have evolved a remarkable variety of structural, physiological, biochemical, and behavioral strategies that enable survival under adverse and fluctuating environmental conditions [1]. However, contemporary global change has accelerated the intensity, frequency, and complexity of environmental perturbations, generating unprecedented selective pressures on living systems worldwide [2]. Anthropogenic climate change, urbanization, habitat fragmentation, salinization, altered nutrient cycles, wildfire intensification, pollution, and anthropogenic acoustic emissions are increasingly transforming ecological systems at local and global scales. In this rapidly changing scenario, understanding how organisms perceive, tolerate, and adapt to environmental stress has become one of the central challenges of modern biology [3]. Importantly, environmental adaptation rarely occurs in response to a single stress factor. Natural systems are increasingly characterized by multifactorial stress conditions in which biological responses emerge through complex interactions among molecular regulation, physiological plasticity, ecological dynamics, and evolutionary processes. Consequently, modern environmental stress biology is progressively moving beyond single-factor analyses toward integrative and multidisciplinary approaches capable of connecting mechanisms across biological scales. The Special Issue (SI) entitled “Adaptation of Living Species to Environmental Stress” was conceived to provide a multidisciplinary platform for investigating these complex responses across molecular, organismal, ecological, and evolutionary levels. The contributions collected in this volume reflect the breadth of this field and demonstrate the remarkable diversity of adaptive strategies across taxa and ecosystems.

## 2. Special Issue Overview

This SI, entitled “Adaptation of Living Species to Environmental Stress”, collected 13 published contributions, including eight original research articles, four review papers, and one correction notice, reflecting the broad scientific interest in emerging perspectives on environmental adaptation. Together, these studies demonstrate how adaptation to environmental stress emerges through interconnected mechanisms operating from the cellular to the ecosystem level. All collected studies have been organized below into separate paragraphs to improve clarity and highlight the multidisciplinary nature of the collection.

## 3. Structural Adaptations and Physical/Acoustic Signals

Sarria-Sarria et al. [4] highlighted a new genus of Andean katydid, Tectucantus, featuring a specialized pronotal structure that acts as a Helmholtz resonator to amplify sound. The researchers tested the hypothesis that the pronotal cavity volume correlates with the carrier frequency of specific calls. The researchers concluded that the pronotal chamber cavity may function as a Helmholtz resonator in all three Tectucantus species examined and, potentially, in other distantly related species employing similar secondary body resonators. Pagano et al. [5] explored how acoustic wave technology—including variations in frequency (Hz), intensity (dB), and treatment duration—influences the growth and physiology of different plant species. The authors highlight the fact that targeted acoustic treatments, such as Plant Acoustic Frequency Technology (PAFT), can significantly enhance key plant growth parameters, including plant height and the number of boll-bearing branches. Furthermore, sound vibrations were found to improve drought tolerance and seed germination rates in crops like rice and cucumber. At a molecular level, specific frequencies (e.g., 250 Hz) were shown to upregulate the expression of certain sound-induced genes.

## 4. Microbial Communities and Urban Pollution

Vetrova et al. [6] characterize antibiotic resistance within the microbial communities of urban dust particles in Moscow. The study reveals a higher prevalence of antibiotic-resistant bacteria (ARB) in zones with greater anthropogenic pollution. Strains resistant to beta-lactams, tetracyclines, and aminoglycosides were detected across all sampled biotopes. Notably, dust deposited on leaves contained bacterial communities with strains resistant to all tested antibiotics, highlighting potential public health risks associated with urban dust circulation. Sazonova et al. [7] compare the taxonomic and functional diversity of bacterial and fungal communities found in atmospheric dust and on the leaf surfaces (phylloplane) of *Tilia* L. across urban zones with varying pollution levels. The results demonstrate that air and phylloplane bacterial communities are more sensitive to environmental pollution than fungal communities, with this effect being particularly pronounced in the phylloplane. The study suggests that these bacterial communities can serve as biological indicators for monitoring air quality.

## 5. Biotechnologies and Extremophiles

Sepe et al. [8] reviewed the vast industrial and environmental applications of extremophiles—microorganisms evolved to survive in Earth’s harshest conditions. Key findings highlight their potential in bioremediation of heavy metals and radioactive waste, the production of second-generation biofuels from lignocellulose, and the development of stable biosensors. Their enzymes, known as extremozymes, are particularly valuable for large-scale biotransformations because they remain robust under extreme temperature and pH levels that would denature standard enzymes.

## 6. Marine Ecosystems and Biological Interactions

Liu et al. [9] analyzed how interspecific competition affects the habitat distribution of the Japanese sardine and chub mackerel. Using species distribution models, the researchers found that interspecific competition significantly alters habitat dynamics, often favoring the expansion of mackerel habitats over those of sardines. The study also identified clear seasonal shifts in optimal habitat areas, highlighting the importance of considering biological interactions alongside environmental factors in fishery management. Palma Esposito et al. [10] investigate the microbiomes of two Maldivian coral species to identify bacteria that could aid in coral restoration against climate change. The authors reported that all coral samples were used to isolate bacteria and assess their bioactivity, leading to the identification of four highly active strains (including a novel species) with antimicrobial, antioxidant, and UV-protective properties. Genomic analysis of these strains revealed functional genes involved in stress response and secondary metabolite production, offering new avenues for both environmental protection and drug discovery.

## 7. Physiological and Molecular Responses to Stress

Komatsu et al. [11] utilized a proteomic approach to investigate how the application of ethanol can mitigate the growth-inhibiting effects of salinity on soybeans. The results indicate that, while salt stress significantly reduces root and hypocotyl growth, ethanol application restores these parameters to levels comparable to control values. Reactive oxygen species enzymes increased under salt stress; notably, mitochondrial ascorbate peroxidase showed further accumulation following ethanol treatment. These outcomes suggest that soybeans were adversely affected by salt stress and recovered with ethanol application via the regulation of cell wall and membrane functions. The systematic review produced by Bizzarri et al. [12] focused on “latent mortality” in conifer forests—delayed tree death that occurs months or years after a fire. A total of 2294 papers published between 2000 and 2024 were identified from Scopus and Web of Science databases. Using the PICO selection method, the authors included 16 relevant studies in the final analysis. The findings revealed that latent mortality is linked to multiple forms of damage and environmental stressors that disrupt hydraulic function and carbon allocation, increasing tree vulnerability to secondary biotic and abiotic stressors. The review concludes that monitoring physiological indicators like sap flow and nonstructural carbohydrates is essential for accurately predicting tree survival and informing post-fire forest management. Additionally, Su et al. [13] examined the role of Heat Shock Protein 70 (HSP70) as a central hub for brain protection during adaptation to high temperatures. The results indicate that HSP70 maintains neuronal homeostasis by coordinating a complex network of autophagy, apoptosis, and inflammation. It promotes the clearance of damaged cellular components through protective autophagy while inhibiting mitochondrial cell death pathways. The study also suggests that HSP70 expression varies by brain region and may form a “heat stress memory” through epigenetic modifications.

## 8. Adaptation, Aging, and Environmental Tolerance

Kallio et al. [14] investigated how extreme climate and urbanization influence biological aging in Russian populations. By applying the Levine PhenoAge model and machine learning, the authors found that residing in extremely cold regions promotes biological age acceleration, which is linked to changes in protein metabolism evidenced by decreased blood albumin levels. Furthermore, the study identifies urbanization as a primary driver of aging; residents of small towns in Central Russia exhibited significantly slower age acceleration compared to those in metropolises or industrial cities. Xing et al. [15] established a histomorphological framework for understanding how the endemic fish *Perca schrenkii* adapts to salinity for aquaculture purposes. The results highlighted that the species investigated exhibits a high innate salinity tolerance, with a critical structural threshold for irreversible damage occurring between 13 and 14 ppt under acute stress. More significantly, the research provides conclusive histological evidence that *Perca schrenkii* can fully acclimate to long-term exposure at salinities up to 7 ppt.

## 9. Conclusions and Future Perspectives

Collectively, the studies included in this Special Issue demonstrate that environmental adaptation is a highly dynamic and multidimensional process requiring integrative perspectives capable of bridging molecular biology, physiology, ecology, evolutionary biology, and environmental sciences. As environmental pressures continue to intensify during the Anthropocene, future research will increasingly depend on interdisciplinary approaches integrating omics technologies, systems biology, ecological modeling, and environmental monitoring. Understanding the mechanisms that regulate stress perception, resilience, and adaptation will be essential not only for biodiversity conservation, but also for sustainable agriculture, ecosystem restoration, climate resilience, and public health. The remarkable diversity of adaptive strategies described in this Special Issue highlights the extraordinary capacity of living systems to respond to environmental change while simultaneously reminding us of the urgent need to preserve the ecological balance upon which such resilience ultimately depends.
